# Supplementary material for: Activity-based protein profiling as a robust method for enzyme identification and screening in extremophilic Archaea
Source: Nat Commun. 2017 May 8;8:15352. doi: 10.1038/ncomms15352 (PMC5424146; doi:10.1038/ncomms15352)
Supplement: Supplementary Information — Supplementary Figures, Supplementary Methods and Supplementary References [file ncomms15352-s1.pdf]

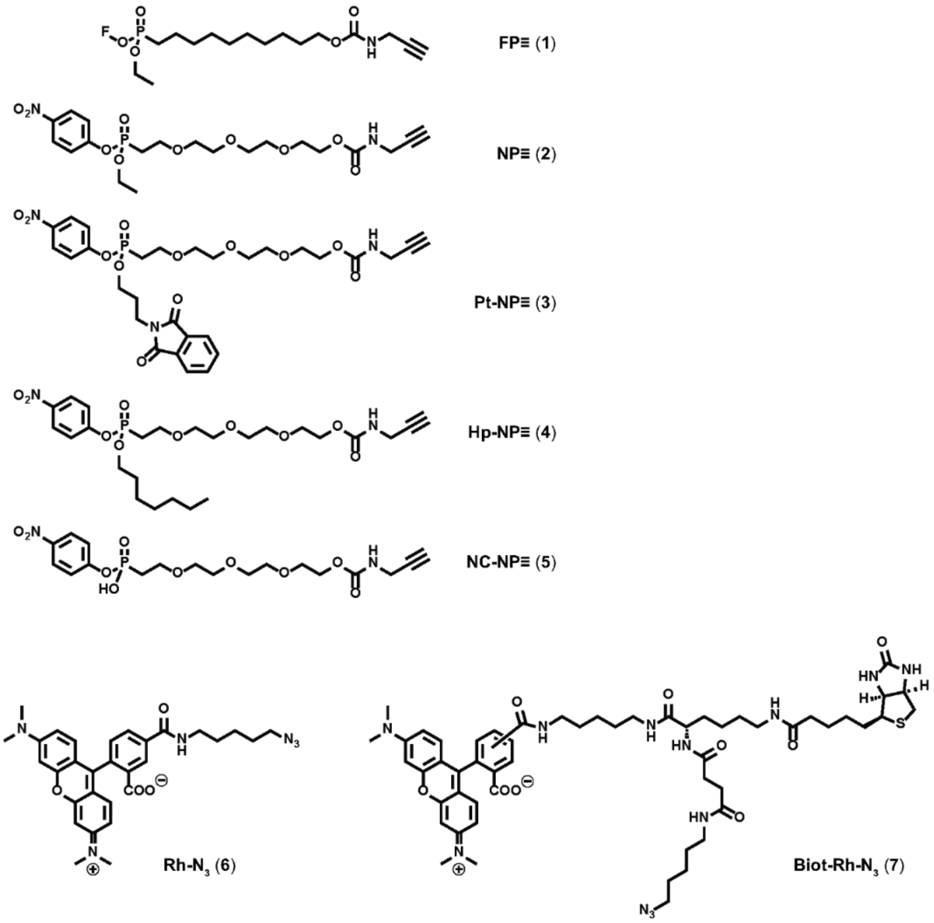

Supplementary Figure 1. Chemical structures of activity-based probes (ABPs) and of click reagents used in this study.

In this study, one fluorophosphonate (**FP≡**, **1**), three nitrophenol phosphonate probes (**NP≡** (**2**), **Pt-NP≡** (**3**) and **Hp-NP≡** (**4**)) as well as one non-reactive negative control compound **NC-NP≡** (**5**) were used. As click reporter, **Rh-N$_3$** (**6**) and **Biot-Rh-N$_3$** (**7**) were used. The synthesis of the activity-based probes is described in the Supplementary Methods section.

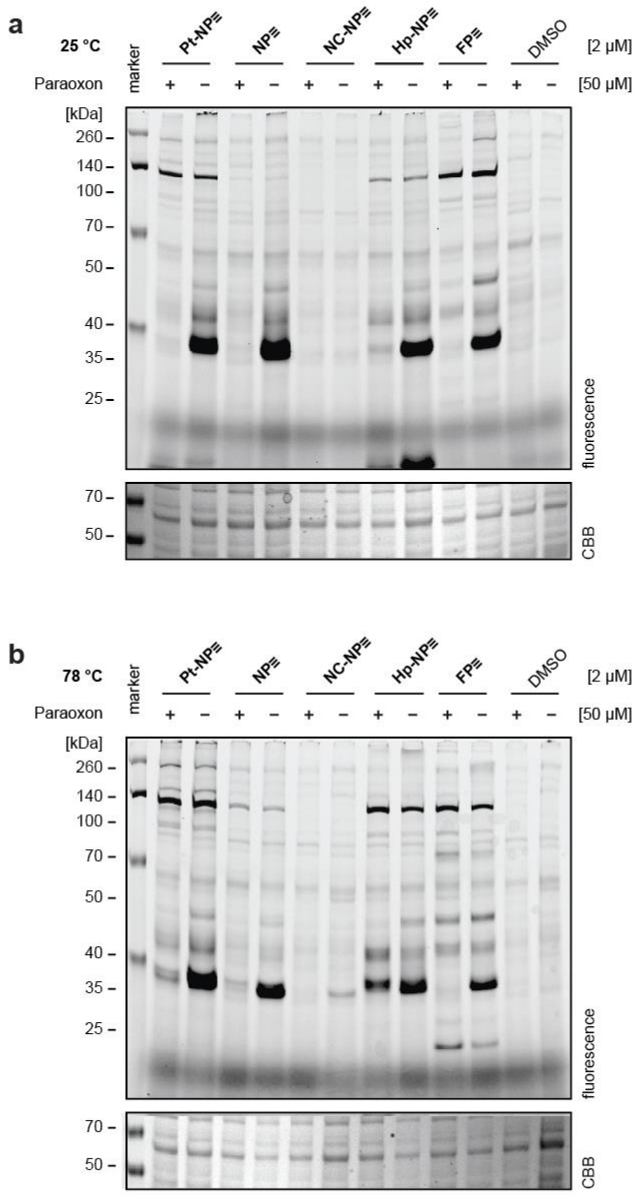

Supplementary Figure 2. *In vitro* ABPP with *S. acidocaldarius* MW001 lysates at 25 °C or 78 °C.

S. acidocaldarius MW001 lysates were treated for 60 min at either 25 °C (**a**) or 78 °C (**b**) with 2 µM of the probe or an equivalent volume of DMSO either without or after pre-incubation with 50 µM paraoxon for 10 min. After 2-step labeling with **Rh-Az** and gel separation labelled proteins were in-gel visualized by detecting fluorescence (Typhoon FLA 9000 scanner). CBB: Coomassie Brilliant Blue staining.

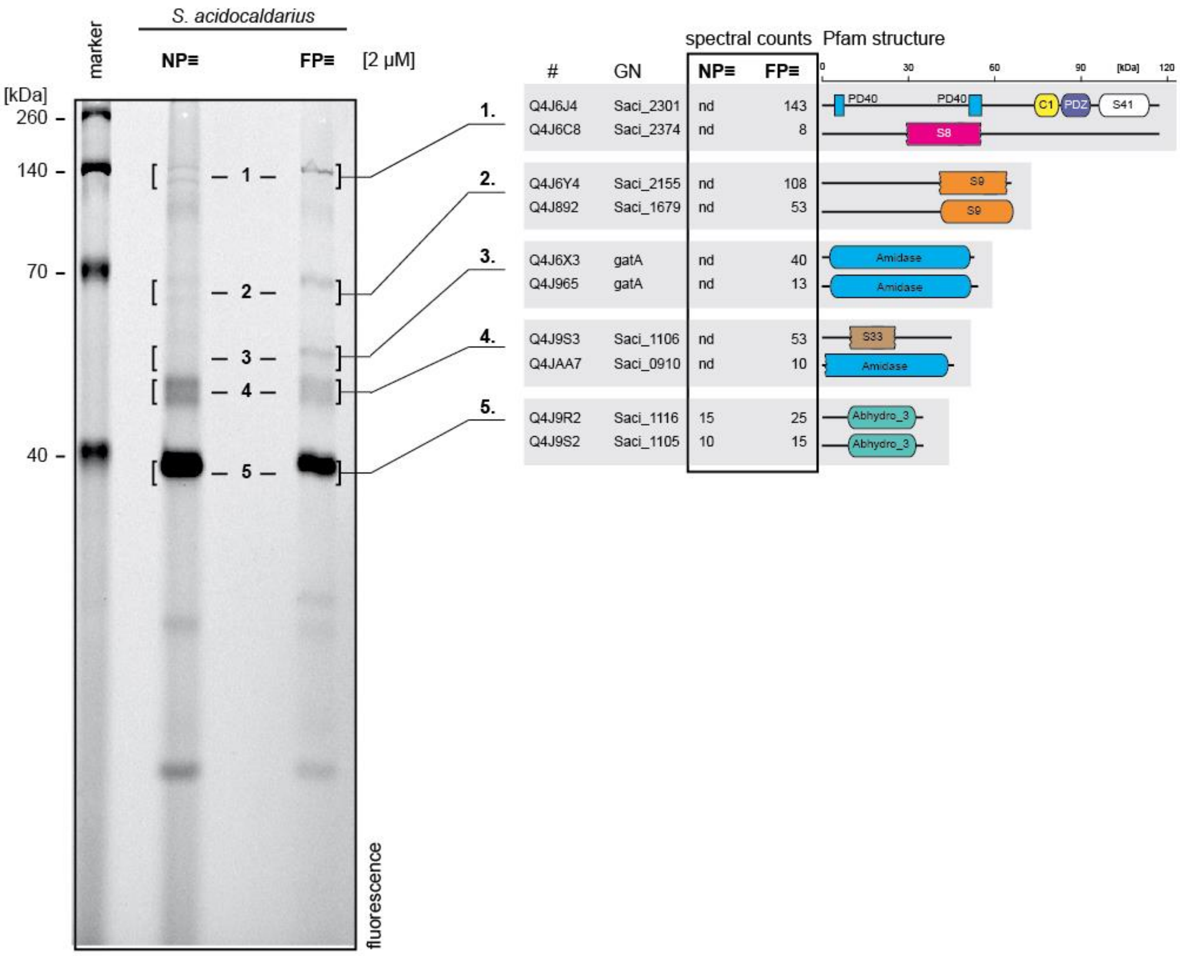

Supplementary Figure 3. Large scale *in vivo* profiling of *S. acidocaldarius* MW001 for MS-based target identification.

S. acidocaldarius growing at 78°C and pH 3.0 was treated for 30 min with 2 µM **NP≡** or **FP≡**. The cells were then collected by centrifugation, washed with culture medium and then lysed with 2% SDS. Labelled proteins were subsequently tagged with **Rh-Biot-Az** (using the 2-step labelling approach) and affinity purified on avidin-agarose beads. The pulled-down proteins were eluted from the beads by boiling in gel loading buffer, separated by SDS PAGE and visualized by in-gel fluorescence detection (Typhoon FLA 9000 scanner). The indicated gel regions were excised from the gel (red brackets), trypsinized and analyzed by LC-MS/MS. The identified serine hydrolases are indicated in the right panel with their corresponding Uniprot ID, gene name (GN), spectral counts and Pfam domain structure.

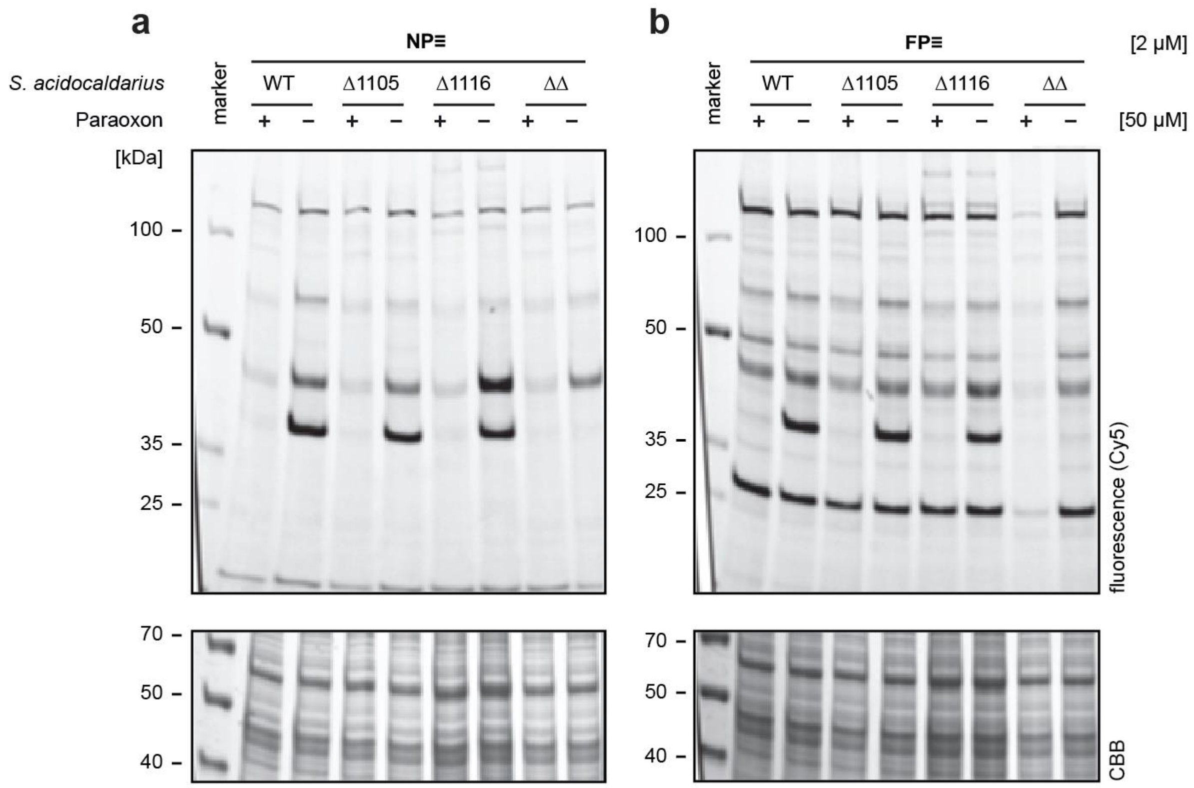

Supplementary Figure 4. *In vivo* labelling of serine hydrolases in *S.acidocaldarius* single and double esterase mutant strains.

Cultures of *S. acidocaldarius* MW001 as well as of an acetylesterase 1116 knock-out mutant Δ1116, a lipase/esterase knock-out mutant Δ1105 and a double-knockout mutant ΔΔ were incubated at 78 °C and pH 3.0 with 2 µM **NP≡** or **FP≡** without or after preincubation with 50 µM paraoxon for 10 min, followed by 2-step labeling with **Rh-Az**, gel separation and in-gel fluorescence detection. CBB = Coomassie Brilliant Blue stain.

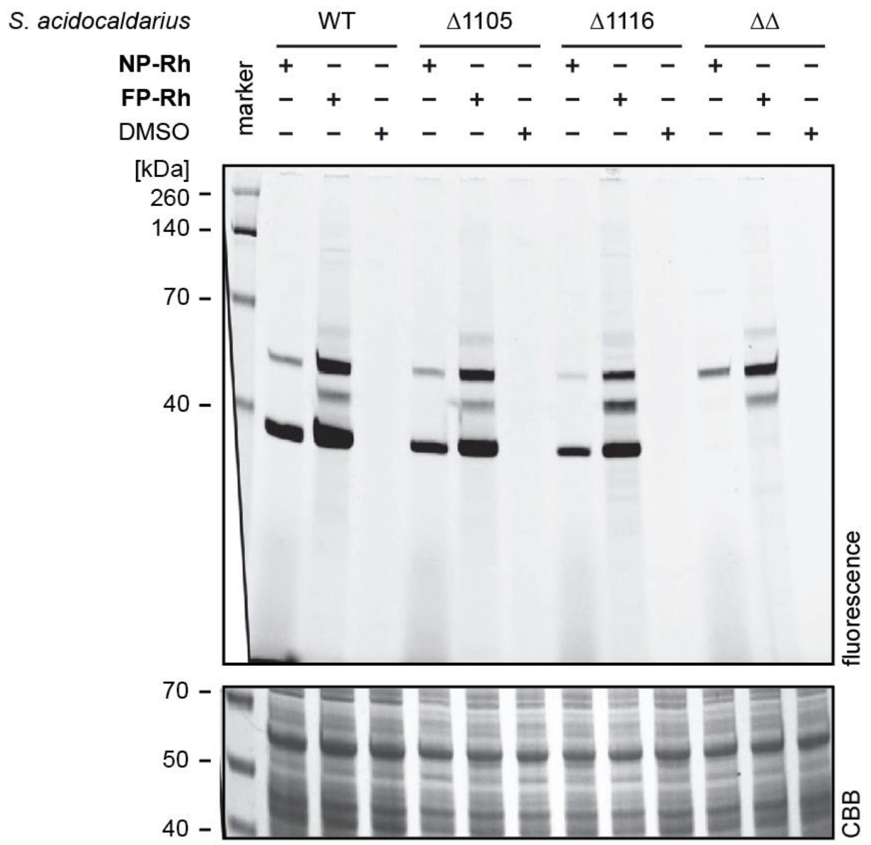

Supplementary Figure 5. *In vitro* labelling of serine hydrolases in *S. acidocaldarius* single and double esterase mutant strains.

Lysates of *S. acidocaldarius* MW001 as well as of an acetylesterase 1116 knock-out mutant Δ1116, a lipase/esterase knock-out mutant Δ1105 and a double-knockout (1105 and 1116) mutant ΔΔ were incubated at 78 °C and pH 3.0 with 2 µM **NP≡** or **FP≡** or DMSO, followed by 2-step labeling with **Rh-Az**, gel separation and in-gel fluorescence detection.

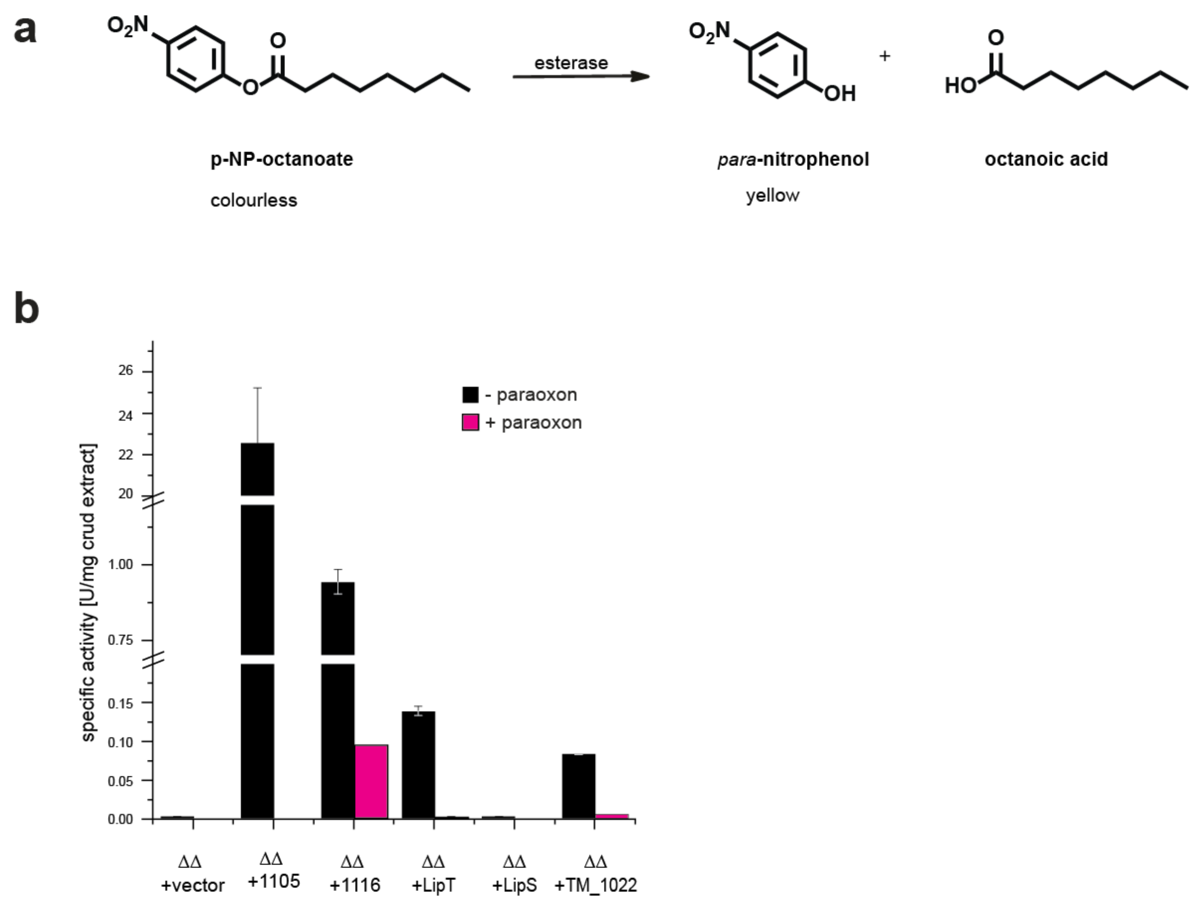

Supplementary Figure 6. Esterase enzyme activity in crude extracts from the constructed esterase deletion strain and cells overexpressing Saci_1105, Saci_1116, LipT, LipS and TM_1022.

(**a**) Chemical reaction underlying the nitrophenol assay. UV spectroscopy is used to detect and quantify liberated *para*-nitrophenol. (**b**) Determination of esterase activity in crude extracts of *S. acidocaldarius* expression cultures. The following strains were tested: The ΔΔ strain (MW903) +empty overexpression vector as negative control (ΔΔ+vector); ΔΔ + pSVAmz_saci1105 (ΔΔ+1105); ΔΔ + pSVA_saci1116 (ΔΔ+1105), ΔΔ + pSVAmZ_p_lipT (ΔΔ+LipT), ΔΔ + pSVAmZ_p_lipS (ΔΔ+LipS) and ΔΔ + pSVAmZ_p_tm1022 (ΔΔ+TM_1022). Esterase activity (black bars) was measured under standard conditions using pNP-octanoate (0.5 mM) and monitoring the release of *p*-nitrophenol at 35 °C (405 nm). For esterase inhibition (red bars) the residual activities were determined after incubation of crude extract with 0.25 mM paraoxon

(60 °C, 1 h). The error bars represent standard deviation of three independent measurements.

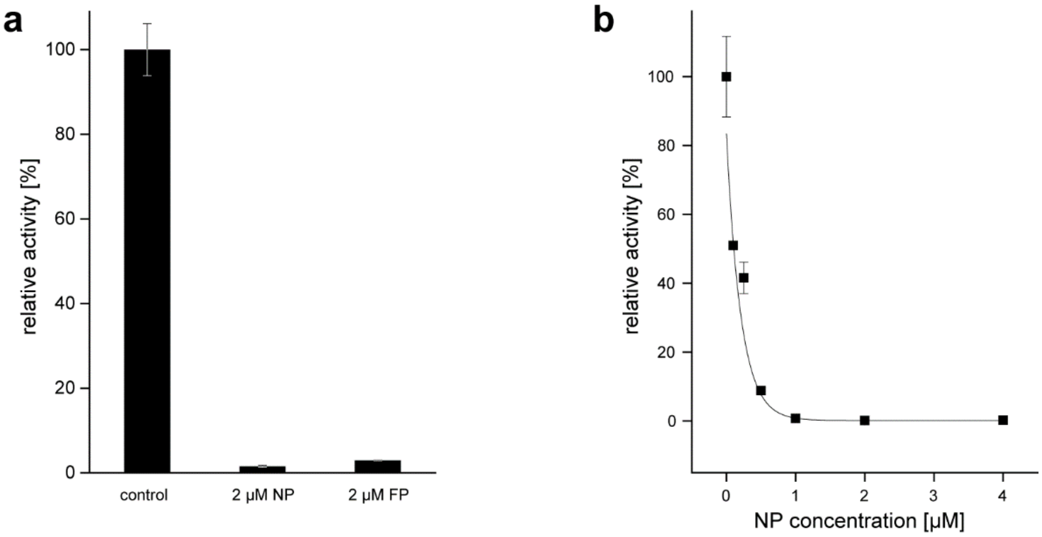

Supplementary Figure 7. Influence of the ABPP probes on esterase activity in crude extracts of *S. acidocaldarius* expression strain ΔΔ + pSVAmz_saci1105 (ΔΔ+1105).

(**a**) Determination of relative esterase activity in crude extracts of ΔΔ+1105 after pre-incubation (30 °C, 1 h) in the presence and absence of 2 µM of **FP≡** and **NP≡** probes. (**b**) Concentration-dependent inhibition of esterase activity in crude extract of strain ΔΔ+1105 after pre-incubation in the presence of the indicated concentrations with the **NP≡** probe (30 °C, 1 h). Residual esterase activity was measured as described in Supplementary Fig. 6. The error bars represent standard deviation of three independent measurements.

Supplementary Methods

Synthesis of ABPs and Click reporters

The **FP≡** probe was synthesized as reported Liu *et al.* [1]. The synthesis of the **NP≡** probe as well as of **Biot-Rh-N$_3$** was published previously by Nickel *et al.* [2].

Pt-NP≡ (3)

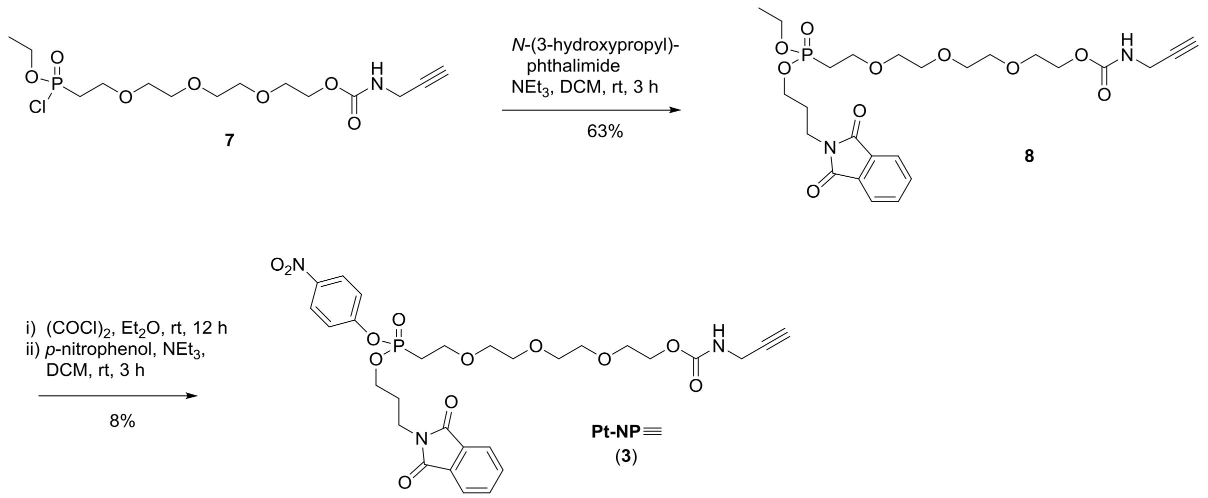

As a starting material for the synthesis of **Pt-NP≡**, a chloro phosphonate derivative **7** (as a common intermediate in the synthesis of **NP≡**) was used. The synthesis of **7** is described in Nickel *et al.* [2].

To a solution of **7** (100 mg, 0.25 mmol) in DCM (10 mL) was added a solution of *N*-(3-hydroxypropyl)-phtalimide (103 mg, 0.5 mmol, 2 eq.) and trietylamine (173 µL, 1.25 mmol, 5 eq.) in DCM (10 mL) and the resulting mixture was stirred at room temperature for 3 h. The solvent was removed in a N_2 stream and the residue was purified by column chromatography ($CHCl_3$/MeOH 19:1) to yield 87 mg (0.16 mmol, 63%) of the desired product **8**.

^{1}H NMR (400 MHz, $CDCl_3$): δ = 7.84 (dd, *J* = 5.4, 3.1 Hz, 2H), 7.72 (dd, *J* = 5.4 Hz, 3.1 Hz, 2H), 4.28-4.20 (m, 2H), 4.13-4.08 (m, 4H), 3.98-3.93 (m, 2H), 3.81 (t, *J* = 7.0

Hz, 2H), 3.77-3.59 (m, 12H), 2.22 (t, *J* = 2.4 Hz, 1H), 2.18-2.01 (m, 4H), 1.30 (t, *J* = 7.0 Hz, 3H); MS (m/z): [M+H]+ calcd. for $C_{25}H_{36}N_2O_{10}P+$, 555.21; found 556.77 [M+H]+ and 578.73 [M+Na]+.

8 (87 mg, 0.16 mmol) was dissolved in DCM (10 mL) and oxalylchloride (274 µL, 3.2 mmol, 20 eq.) was added. The resulting mixture was stirred overnight at room temperature. The solvent was removed in a N_2 stream and the residue was dried in high vacuum. The residue was taken up in DCM (10 mL) and a solution of nitrophenol (45 mg, 0.32 mmol, 2 eq.) and triethylamine (111 µL, 0.8 mmol, 5 eq.) in DCM (10 mL) was added. The resulting mixture was stirred at room temperature for 3 h. The solvent was removed in a N_2 stream and the residue was first purified by a silica gel column chromatography ($CHCl_3$/MeOH 19:1) and then again by reverse chromatography on a self-packed C18 column (60% aq. acetonitrile) to yield 8 mg (0.012 mmol, 8%) of the desired product **Pt-NP≡** (**3**).

^{1}H NMR (400 MHz, $CDCl_3$): δ = 8.22 (d, *J* = 8.3 Hz, 2H), 7.85-7.83 (m, 2H), 7.74-7.72 (m, 2H), 7.40 (d, *J* = 8.5 Hz, 2H), 4.27-3.62 (m, 20H), 2.36-2.07 (m, 5H); MS (m/z): $[M+H]^+$ calcd. for $C_{29}H_{35}N_3O_{12}P^+$, 648.20; found 647.03 $[M+H]^+$ and 669.03 $[M+Na]^+$.

Hp-NP≡ (4)

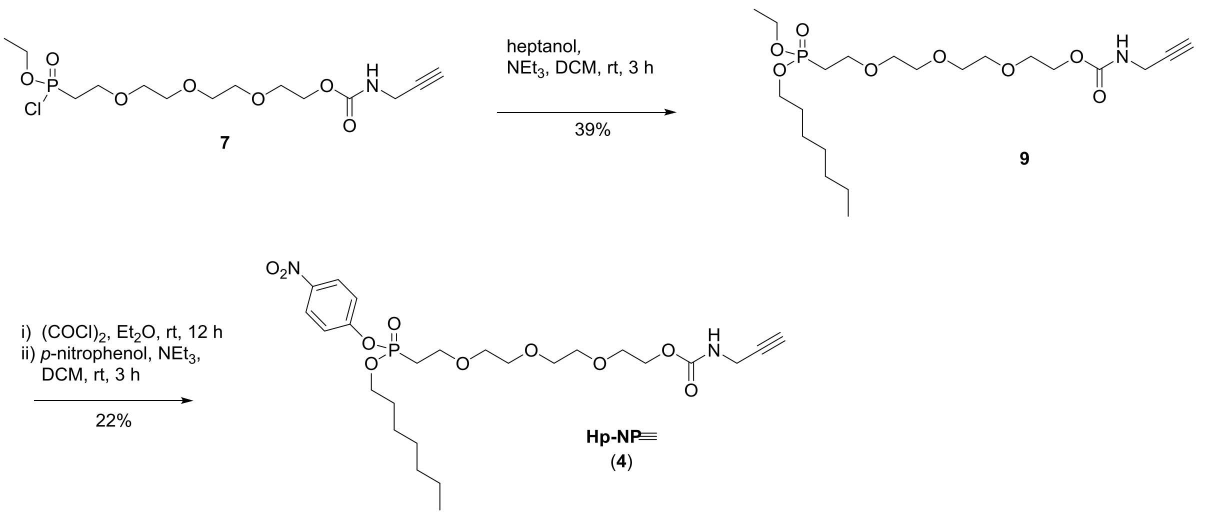

To a solution of **7** (100 mg, 0.25 mmol) in DCM (10 mL) was added a solution of heptanol (71 µL, 0.5 mmol, 2 eq.) and trietylamine (173 µL, 1.25 mmol, 5 eq.) in DCM

(10 mL) and the resulting mixture was stirred at room temperature for 3 h. The organic phase was washed with water, dried over Na_2SO_4 and evaporated to dryness. The residue was purified by column chromatography (DCM/MeOH 9:1) to yield 45 mg (0.10 mmol, 39%) of the desired product **9**.

9 (45 mg, 0.10 mmol) was dissolved in DCM (10 mL) and oxalylchloride (172 µL, 2.0 mmol, 20 eq.) was added. The resulting mixture was stirred overnight at room temperature. The solvent was removed in a N_2 stream and the residue was dried in high vacuum. The residue was taken up in DCM (10 mL) and a solution of nitrophenol (28 mg, 0.2 mmol, 2 eq.) and triethylamine (69 µL, 0.50 mmol, 5 eq.) in DCM (10 mL) was added. The resulting mixture was stirred at room temperature for 3 h. The solution was washed with water, dried over Na_2SO_4 and reduced to dryness. The residue was purified by column chromatography (DCM/MeOH 50:1) to yield 20 mg (0.04 mmol, 40 %) of the desired product **Hp-NP≡** (**4**).

^{1}H NMR (400 MHz, $CDCl_3$): δ = 8.34 (d, *J* = 9.0 Hz, 2H), 7.55-7.49 (m, 2H), 4.52-4.17 (m, 4H), 3.93-3.76 (m, 4H), 3.74-3.64 (m, 10H), 2.60 (s, 1H), 2.47-2.42 (m, 2H), 1.78-1.61 (m, 2H), 1.39-1.31 (m, 8H), 0.93 (t, *J* = 6.9 Hz, 3H).

MS (m/z): $[M+H]^+$ calcd. for $C_{25}H_{40}N_2O_{10}P^+$, 559.24; found 559.02 $[M+H]^+$ and 581.24 $[M+Na]^+$.

Supplementary References

1. Liu Y, Patricelli MP, Cravatt BF (**1999**) Activity-based protein profiling: the serine hydrolases. Proc. Natl. Acad. Sci. USA 96: 14694-14699.
2. Nickel S, Kaschani F, Colby T, van der Hoorn RA, Kaiser M (**2012**) A para-nitrophenol phosphonate probe labels distinct serine hydrolases of Arabidopsis. Bioorg. Med. Chem. 20: 601-606.
