# Supplementary material for: Activity-based protein profiling as a robust method for enzyme identification and screening in extremophilic Archaea
Source: Nat Commun. 2017 May 8;8:15352. doi: 10.1038/ncomms15352 (PMC5424146; doi:10.1038/ncomms15352)
Supplement: Supplementary Data 3 — List of strains, primers and plasmids used in this study. [file ncomms15352-s4.docx]

| **Strains, Primers and plasmids** | **Relevant characteristics** | | **Source or reference** |
| --- | --- | --- | --- |
| ***Sulfolobus acidocaldarius*** | | | |
| *Saci* MW 001 | Wild-type, *pyrE*- (a 322 bp deletion in the *pyrE* gene) | | Wagner et al., 2012 |
| *Saci* MW 901 | MW001, Δ*Saci*_1105, *pyrE* | | This study |
| *Saci* MW 902 | MW00,1 Δ*Saci*_1116, *pyrE* | | This study |
| *Saci* MW 903 | MW001, Δ*Saci*_1105/Δ*Saci*_1116, *pyrE* | | This study |
| ***Escherichia* *coli*** | | | |
| K12 DH5a | l2 f80d/lacZDM15 D(lacZYA-argF)U169 recA1 endA1 hsdR17 (rK2 mK1) supE44 thi-1 gyrA relA1 | | Invitrogen |
| Rosetta DE3 pLysS | F^-^ *ompT hsdS*_B_(r_B_^-^ m_B_^-^) *gal dcm* (DE3) pRARE (Cam^R^) | | Novagen |
| ER1821 | FglnV44 e14^-^ (McrA^-^ ) rfbD1? relA1? endA1 spoT1? thi-1 Δ(mcrC-mrr) 114::IS10 (containing pM.EsaBC4I plasmid) | | NEB |
| ***Haloferax volcanii*** | | | |
| H26 | ΔpyrE2 | | Allers et al., 2004 |
| **Primers** |  | **Target Vector** |  |
| Saci_1105_*NheI_For* | AAAGCTAGCATGCCGTTGGATCCTGAAG | pET24a | This study |
| Saci_1105_*XhoI_Rev* | AAACTCGAGCTTAAAAATGTCCTTAAGTAGTG | pET24a | This study |
| Saci_1105_FP | CGGAGGTGTCCTTAAGTTTAG | pSVAmZ-SH10 | This study |
| Saci_1105_RP | GTGGATCAAAATTGTATCC | pSVAmZ-SH10 | This study |
| Saci_1116_*NheI_For* | AAAGCTAGCATGCCTTTAGATCCAACCATA | pET24a | This study |
| Saci_1116_*XhoI_Rev* | AAACTCGAGAAAACTGTTGTAGAATACTCG | pET24a | This study |
| Saci_1116_*NcoI_For* | TATCCATGGCTCCTTTAGATCCAACCATAAAG | pSVA1551 | This study |
| Saci_1116_*BamHI_Rev* | ATAGGATCCAAAACTGTTGTAGAATACTCGTCTC | pSVA1551 | This study |
| Saci_1116_*NcoI-site-del_For* | GTAATCCACGGCTTCGTCTCCTTCTTCCC | pSVA1551 | This study |
| Saci_1116_*NcoI-site-del_Rev* | GACGAAGCCGTGGATTACGTTATTAAACCTGAC | pSVA1551 | This study |
| Saci_1116_SacII_For | GGTCCGCGGGTAAATGTCCTAAAGGTAGATG | pSVAmZ-SH10 | This study |
| Saci_1116_BamHI_Rev | GGTGGATCCAGAATTACAGCTTTCATAGTTC | pSVAmZ-SH10 | This study |
| *lipT* -*Nhe*I_For | AATGCTAGCATGCGGCGGTTACTAGCC | pSVA2301 | This study |
| *lipT*-short-*Not*I_Stop_Rev | AATGCGGCCGCTCAGCGCACCCTAGGCGCCGCCT | pSVA2301 | This study |
| LipS­_NheI_For | TAAGCTAGCATGAGCCCGAAAAGCAGGA | pSVA2301 | This study |
| *lipS* -*Not*I_Stop_Rev | TAAGCGGCCGCTCAGCTGTGCTTCCGGATGAACGC | pSVA2301 | This study |
| Tm1022_Sac*II*_F | GGTCCGCGGGAACGGCTATCAATGTAGGT | pSVAmZ-SH10 | This study |
| Tm1022_Bam*HI*_RII | GGTGGATCCACGAGACCTATCTGAGC | pSVAmZ-SH10 | This study |
| Tm1160_Sac*II*_F1 | GGTCCGCGGGATCATAGCAGCTTTCATAC | pSVAmZ-SH10 | This study |
| Tm1160_Bam*HI*_R1 | GCTGGATCCTATCATCACCGAGCCCTG | pSVAmZ-SH10 | This study |
| Pf2001_Sac*II*_F1 | GGTCCGCGGGAAGCCAGCGTCCGA | pSVAmZ-SH10 | This study |
| Pf2001_Bam*HI*_RII | GGTGGATCCAACTACGGTGGGGTGAA | pSVAmZ-SH10 | This study |
| mod_BamHI_del_1_fw | TATCGGCCGCAGATGTGTATAAGAGACAGAC | pSVA1551 construction | This study |
| mod_BamHI_del_2_fw | AATGAAACACGGGGTTACCGATCCAGACAAGATATTG | pSVA1551 construction | This study |
| mod_BamHI_del_2_rev | CAATATCTTGTCTGGATCGGTAACCCCGTGTTTCATT | pSVA1551 construction | This study |
| mod_BamHI_del_3_fw | CACACCAATTCCCGCCGGACCCTAACTTTTTCGAT | pSVA1551 construction | This study |
| mod_BamHI_del_3_rev | ATCGAAAAAGTTAGGGTCCGGCGGGAATTGGTGTG | pSVA1551 construction | This study |
| mod_BamHI_del_4_fw | TCCATATGTTAATCCTGGTCGGTTCCGCAAAATTTTAGTTATAAG | pSVA1551 construction | This study |
| mod_BamHI_del_4_rev | CTTATAACTAAAATTTTGCGGAACCGACCAGGATTAACATATGGA | pSVA1551 construction | This study |
| mod_BamHI_del_5_fw | GTTAATCCTACTTTACATGGGTTCCCATATGAGTGATCTGAAG | pSVA1551 construction | This study |
| mod_BamHI_del_5_rev | CTTCAGATCACTCATATGGGAACCCATGTAAAGTAGGATTAAC | pSVA1551 construction | This study |
| mod_BamHI_del_rev | TTACCCGGGAGCGTGGAAAAATAGTAG | pSVA1551 construction | This study |
| mod_MCS_del_fw | CTTCAATAATCGGTACCTCGTGTAGATTTTCC | pSVA1551 construction | This study |
| mod_MCS_del_rev | CACGAGGTACCGATTATTGAAGCATTTATCAGG | pSVA1551 construction | This study |
| mod_MCS_ins_fw | TGTCCATGGTAAATGGATCCGAGAACCTGTACTTCCAAGGAGGTTCGCTCGAGTGGAGTCATCCACAATTTGAG | pSVA1551 construction | This study |
| mod_MCS_ins_rev | ATAGCGGCCGCAATCTAATGAAAATGAGATTAG | pSVA1551 construction | This study |
| mod_Size_fw | ATTGCGGCCGCTTTCACACGGGTAATACTGAAAAATCTAC | pSVAmZ-SH10 construction | This study |
| mod_Size_rev | TATCCCGGGTTAGATAAATTCAAATTTTTGTTGTAAG | pSVAmZ-SH10 construction | This study |
| mod_NdeI_del_fw | GAAGAGAAACATTTGTCACTGGATTGATGAAGAG | pSVAmZ-SH10 construction | This study |
| mod_NdeI_del_rev | CCAGTGACAAATGTTTCTCTTCTCGCACAAC | pSVAmZ-SH10 construction | This study |
| mod_lacZ_fw | TTACCATGGCTCATATGCGCCCAATACGCAAACCGC | pSVAmZ-SH10 construction | This study |
| mod_lacZ_rev | CTCGGATCCGTCGACGCTAGCGACGTCTTAATGCGCCGCTACGGCAAAGCGCCATTCGCCATTC | pSVAmZ-SH10 construction | This study |
| ko_1105_fw | GATGGGCCCTAAGTGCTGGAGGTAATTTG | pSVA486 construction | This study |
| ko_1105_rev | GATCTCGAGTTACTTAAAAATGTCCTTAAGTAG | pSVA486 construction | This study |
| ko_1105_up_fw | GATCCTAGGGCCTCTTGAACCTAGAATC | pSVA486 construction | This study |
| ko_1105_up_rev | CTTAAAAATGTCCAACGGCATAACATTTAGTTTTAG | pSVA486 construction | This study |
| ko_1105_down_fw | GTTATGCCGTTGGACATTTTTAAGTAAAAGAAACATTG | pSVA486 construction | This study |
| ko_1105_down_rev | GATCGGCCGTTTCATTGCACCATACATC | pSVA486 construction | This study |
| ko_1116_fw | GATGGGCCCGGAAAGCCTAGAGGGTAAGC | pSVA488 construction | This study |
| ko_1116_rev | GATCTCGAGTCAAAAACTGTTGTAGAATAC | pSVA488 construction | This study |
| ko_1116_up_fw | GATCCTAGGCGGAGGAGTTTACTTTGTC | pSVA488 construction | This study |
| ko_1116_up_rev | CAAAAACTGTTGTGGATCTAAAGGCATAATCTATTTATAC | pSVA488 construction | This study |
| ko_1116_down_fw | CCTTTAGATCCACAACAGTTTTTGAATACAAAATTTTAATTATTTTTG | pSVA488 construction | This study |
| ko_1116_down_rev | GATCGGCCGTCTTAAGCCCTCTGTAAATC | pSVA488 construction | This study |
| ex_lipT_fw NcoI | TATCCATGGCTCGGCGGTTACTAGCCTTGC | pSVA1431 | This study |
| ex_lipT_rev NotI | AATGCGGCCGCTCAGCGCACCCTAGGCGCCGCCT | pSVA1431 | This study |
| ex_lipS_fw NcoI | TATCCATGGCTAGCCCGAAAAGCAGGAACTG | pSVA1431 | This study |
| ex_lipS_NcoI_del_fw | CACCGCCCATTGAGAGGCCGGTCATGAAGAG | pSVA1431 | This study |
| ex_lipS_NcoI_del_rev | GCCTCTCAATGGGCGGTGCCCTGACGG | pSVA1431 | This study |
| ex_lipS_rev NotI | TAAGCGGCCGCTCAGCTGTGCTTCCGGATGAACGC | pSVA1431 | This study |
| ex_1116_fw | TATCCATGGCTCCTTTAGATCCAACCATAAAG | pSVA1551 | This study |
| ex_1116_NcoI_del_fw | GTAATCCACGGCTTCGTCTCCTTCTTCCC | pSVA1551 | This study |
| ex_1116_NcoI_del_rev | GACGAAGCCGTGGATTACGTTATTAAACCTGAC | pSVA1551 | This study |
| ex_1116_rev | ATAGGATCCAAAACTGTTGTAGAATACTCGTCTC | pSVA1551 | This study |
| **Plasmids** |  |  |  |
| pET24a | Kan^r^, lac op, pBR322 ori, T7, lacI, f1 ori, T7 tag, His, t7 term | | Novagen |
| pMZ1 | pre vector | | Zolghadr et al., 2007 |
| pSVA1431 | expression plasmid | | Wagner et al., 2014 |
| pSVA1551 | modified pSVA1431, see material and methods | | This study |
| pSVA431 | Gene targeting plasmid, pyrEFSSO and lacSSSO cassette; double crossover method | | Wagner *et al.*, 2012 |
| pSVA485 | *saci_1105* in-frame deletion prevector, *saci_1105* cloned into pSVA431 with *Apa*I, *Xho*I | | This study |
| pSVA486 | *saci_1105* in-frame deletion, *saci_1105* in-frame deletion fragment cloned into pSVA485 with *Avr*II, *Eag*I | | This study |
| pSVA487 | *saci_1116* in-frame deletion prevector, *saci_1105* cloned into pSVA431 with *Apa*I, *Xho*I | | This study |
| pSVA488 | *saci_1116* in-frame deletion, *saci_1116* in-frame deletion fragment cloned into pSVA485 with *Avr*II, *Eag*I | | This study |
| pSVA1450 | expression plasmid | | Wagner et al., 2014 |
| pSVA2301 | modified pSVA1450, see material and methods | | This study |
| pSVAmZ-SH10 | modified pSVA1551, see material and methods | | This study |
| pET24a-saci_1105 | pET24a, s*aci_1105_6xHis_* cloned with *Nhe*I and *Xho*I | | This study |
| pET24a-saci_1116 | pET24a, *saci_1116_6xHis_* cloned with *Nhe*I and *Xho*I | | This study |
| pSVA-lipT | pSVA2301, *lipT* cloned with *Nhe*I and *Not*I with stop codon | | This study |
| pSVA-lipS | pSVA2301, *lipS* cloned with *Nhe*I and *Not*I with stop codon | | This study |
| pSVA-saci_1116 | pSVA1551, *saci_1116_6xHis_ cloned with NdeI and XhoI* | | This study |
| pSVAmZ-saci_1105 | pSVAmZ-SH10, *saci_1105*_6xHis_ with *Nco*I and *Xho*I | | This study |
| pSVAmZ-p-tm1022 | pSVAmZ-SH10, *tm1022* with 471bp promotor region, cloned with *Sac*II, *Bam*HI | | This study |
| pSVAmZ-p-tm1160 | pSVAmZ-SH10, *tm1160* with 421bp promotor region, cloned with *Sac*II, *Bam*HI | | This study |
| pSVAmZ-p-pf2001 | pSVAmZ-SH10, *pf2001* with 371bp promotor region, cloned with *Sac*II, *Bam*HI | | This study |
